# Moral Foundations and Obesity: The Role of Binding vs. Individualizing Foundations in Shaping Weight Stigma

**DOI:** 10.5334/irsp.1068

**Published:** 2025-07-18

**Authors:** Cristian Catena-Fernández, Alejandro Magallares

**Affiliations:** 1 Universidad Nacional de Educación a Distancia (UNED), ES

**Keywords:** moral foundations, obesity, anti-fat, weight stigma

## Abstract

Weight stigma significantly affects the quality of life for individuals with obesity in Western societies. While previous research has used moral foundations theory to predict attitudes toward stigmatized groups, such as the poor, immigrants, and sexual minorities, its application to weight-related stigma remains underexplored. This research explores the relationship between moral foundations, the moralization of obesity, and weight stigma in an underrepresented European context. In a pre-registered correlational study (Study 1), we found that binding and individualizing foundations differentially predicted the moralization of obesity and weight stigma. A follow-up pre-registered experiment (Study 2) suggested that highlighting the societal benefits of purity, a binding moral foundation, over care, an individualizing moral foundation, may increase the moralization of obesity and heighten weight stigma. These findings contribute to deepening understanding of the moral roots of weight stigma and underscore the importance of considering moral values in efforts to mitigate it.

As obesity rates continue to escalate in Western societies, understanding its broader social and psychological impacts is crucial. While obesity itself is a major health concern, the stigma attached to it exacerbates the challenges faced by individuals with obesity. Weight stigma infiltrates various domains, including healthcare, workplaces, educational contexts, interpersonal relations, and mass media, significantly diminishing the quality of life for individuals with obesity both in North America and Western Europe (Gaspar et al. 2022; Gracia-Arnaiz 2013; Puhl et al. 2021; Rubino et al. 2020). Importantly, beyond impairing quality of life, weight stigma has also been linked to adverse physical and mental health outcomes (e.g., physiological stress and disordered eating; Major et al. 2017). The present research examines the relationship between moral psychology and weight stigma, investigating how different moral framings shape negative attitudes toward individuals with obesity.

## The moral roots of weight stigma

Obesity has long been framed as a moral failing (Woodhouse 2008). While social psychology has only recently examined weight stigma through moral judgment, emerging evidence suggests moral frameworks play a key role. For instance, some studies indicate that children associate weight with intrinsic character (essentialist moral view), fostering strong biases (Carvalho et al. 2021; Peretz-Lange et al. 2024). This perspective frames weight as a marker of virtue or failure, making obesity a moral issue. Similarly, research on social dominance orientation shows that those favoring societal hierarchies exhibit stronger weight biases, viewing individuals with obesity as lower status (Elison & Çiftçi 2015; Magallares 2014; O’Brien et al. 2013). These findings suggest weight stigma extends beyond social prejudice, deeply rooted in moral reasoning, making perspectives like moral foundations theory essential for understanding the moral roots of weight stigma.

According to moral foundations theory (Graham et al. 2009), there are five primary moral foundations: *Harm/care*, which fosters compassion and concern for others; *Fairness/reciprocity*, which underpins justice and equitable treatment; *Ingroup/loyalty*, which emphasizes group allegiance; *Authority/respect*, which values social order and tradition; and *Purity/sanctity*, which regards both physical and metaphysical cleanliness as sacred (for a review see Graham et al. 2009).^1^ These foundations are grouped as *individualizing foundations* (harm/care and fairness/reciprocity), focused on well-being and rights, and *binding foundations* (ingroup/loyalty, authority/respect, and purity/sanctity), emphasizing social cohesion (Haidt 2012; Graham et al. 2013).

Research shows that these moral foundations are differentially associated with social attitudes and prejudices. Individuals who endorse binding foundations are more likely to express intergroup hostility and negative attitudes toward stigmatized groups, whereas those who prioritize individualizing foundations tend to exhibit more inclusive and egalitarian attitudes (Argüello-Gutiérrez et al. 2024; Barnett et al. 2020; Kugler et al. 2014; Low & Wui, 2016; Monroe & Plant, 2019; Van de Vyver et al. 2016). For instance, Low and Wui (2016) found that endorsement of individualizing foundations predicted more favorable views toward the poor, while binding foundations were associated with blaming the poor for their condition. Similarly, Barnett et al. (2020) showed that homonegative attitudes were positively associated with purity and authority concerns, and negatively associated with harm and fairness, highlighting how specific moral concerns can underline and reinforce stigmatizing beliefs toward sexual minorities.

Building on this framework, we proposed that moral foundations can similarly predict attitudes towards obesity. Specifically, we hypothesized that endorsement of binding foundations would be positively associated with higher levels of weight stigma whereas endorsement of individualizing foundations would be negatively associated with it. In Study 1, we examined these broad dimensions to capture the full spectrum of moral orientations associated with weight stigma and identify general patterns of association. Based on these findings, in Study 2 we focused on the care and purity foundations as prototypical exemplars of the individualizing and binding dimensions, respectively (Monroe & Plant 2019; Mooijman et al. 2018; Wagemans et al. 2018), given their particular importance for understanding how obesity becomes moralized and stigmatized.

## Care and purity foundations

The purity foundation has been associated with ideals of bodily control, self-discipline, and sanctity, which can be perceived as violated by individuals with obesity (Clifford & Wendell 2016; Gaspar et al. 2022; Haidt 2012; Lieberman et al. 2012; Masicampo et al. 2014; Mooijman et al. 2018; Ringel & Ditto 2019; Rozin 1999). Previous research indicates that moral concerns regarding physical purity and contamination frequently extend to how others’ bodies are evaluated, particularly in Western societies where thinness is not only aesthetic but moralized (Gaspar et al. 2022; Rozin 1999). For instance, individuals who strongly endorse purity may perceive obesity as a violation of bodily norms, evoking disgust and moral condemnation (Clifford & Wendell 2016; Lieberman et al. 2012). In line with this, Masicampo et al. (2014) showed that disgust, a core emotional response linked to purity, amplifies moral judgments specifically within the domain of purity. Because individuals with obesity are often perceived as violating cultural ideals of self-control and bodily integrity, they may be judged more harshly in moral terms when purity concerns are salient (Mooijman et al. 2018).

In contrast, the care foundation promotes sensitivity to harm and suffering, encouraging more compassionate and non-blaming attitudes. Individuals high in care values may be more inclined to interpret obesity through structural or psychological explanations—such as socioeconomic disadvantage, trauma, or mental health issues—rather than seeing it as a personal failing (Crandall 1994; Haidt 2012). Research by Horberg et al. (2009) demonstrates that emotions linked to care, such as empathy and sympathy, reduce moral condemnation by framing transgressions or challenges in a broader context of human vulnerability.

Thus, care and purity may exert opposing effects on stigma by shaping moral attributions in divergent directions. Whereas purity may reinforce internal attributions and moralize obesity as a failure of discipline or self-respect, care may foster external attributions and reduce the impulse to moralize (Gaspar et al. 2022; Ringel & Ditto 2019). These foundations may therefore influence the degree to which individuals moralize obesity—and, in turn, the extent to which they stigmatize individuals with obesity.

## The moralization of obesity and weight stigma

We further propose that moral foundations not only predict weight stigma but also contribute to the moralization of obesity. Moralization refers to the psychological process whereby a condition transitions from being perceived primarily as a matter of health concern into one explicitly viewed as morally objectionable and ethically condemnable (Rozin 1999). According to Ringel and Ditto (2019), moralization of obesity involves explicitly associating obesity with moral faults such as lack of self-control, laziness, or personal irresponsibility, implying moral blame directed at the condition itself.

In contrast, weight stigma—measured by scales like the Antifat Attitudes Scale (Crandall 1994)—reflects negative evaluations toward individuals with obesity. These attitudes encompass social dislike, interpersonal rejection, fear of becoming fat oneself, and beliefs about personal responsibility for weight. While such attitudes can involve moral judgment, they are primarily rooted in prejudice rather than explicit ethical condemnation. Although moralization and weight stigma are conceptually intertwined (Yang et al. 2007), we follow prior works (e.g., Ringel & Ditto 2019; Rozin 1999) in treating moralization as a distinct cognitive process—specifically, as an antecedent of stigma that helps explain how individuals come to morally condemn obesity, thereby amplifying negative attitudes toward those with the condition.

Empirical findings from Ringel and Ditto (2019) further clarify that moralization uniquely intensifies weight stigma by eliciting greater perceived controllability and blameworthiness of obesity, more significant support for discriminatory behaviors, and stronger moral emotions (such as disgust). Thus, moralization is a potent mechanism amplifying stigma precisely because it positions obesity explicitly within the domain of moral wrongdoing.

Building on this, we also aimed to test whether the indirect effects of moral foundations of care and purity would be mediated by moralization of obesity. Research linking obesity to disgust (Clifford & Wendell 2016; Lieberman et al. 2012; Park et al. 2007) suggests that obesity is often seen as a violation of bodily purity, making it particularly susceptible to moral condemnation (Haidt 2012; Wagemans et al. 2018). Conversely, individuals prioritizing care may interpret obesity with compassion, acknowledging socioeconomic and psychological factors rather than attributing it solely to personal failure (Graham et al. 2011; Haidt 2012; Masicampo et al. 2014).

## Overview of the present research

We conducted two studies in Spain, where obesity is a growing public health concern. The *Informe Anual del Sistema Nacional de Salud 2022* [Annual Report of the National Health System] reports that 16% of adults are classified as obese, with prevalence rising over the past 30 years (Feijoo et al. 2024). While obesity poses significant health risks, we argue that weight stigma is both a consequence and a contributing factor, exacerbating these challenges. Stigma against individuals with obesity in Spain is well documented (Gaspar et al. 2022; Gracia-Arnaiz 2013; Magallares et al. 2024), highlighting the need to examine societal attitudes and moral judgments toward obesity.

Study 1 examined how endorsement of moral foundations relates to moralization of obesity and weight stigma. Our main hypothesis was that greater endorsement of the binding foundations would be associated with higher levels of moralization of obesity and weight stigma. Conversely, greater endorsement of the individualizing foundations would be associated with lower levels of moralization of obesity and weight stigma. Additionally, we explored the relationship between moral foundations and two exploratory outcomes: moral positive stereotypes toward individuals with obesity and willingness to engage in collective action in support for the rights of individuals with obesity. Morality is a central component of group impression formation, embedded within the broader dimension of warmth (Abele et al. 2021; Brambilla et al. 2021; Leach et al. 2007). In this sense, moral stereotypes may serve as a mechanism for morally devaluing stigmatized groups (Brambilla et al. 2021). Moreover, moral foundations have been associated with intentions to engage in collective action on behalf of migrants, whereas binding foundations are negatively associated (Argüello-Gutiérrez et al. 2024; Milesi 2017).

Study 2 focuses on the causal impact of two specific moral foundations: purity and care. The manipulation of these foundations allows us to better explore their causal impact on weight stigma. Previous studies show that moral foundation activation influences social attitudes (e.g., Argüello-Gutiérrez et al. 2024; Day et al. 2014; Winterich et al. 2012). For instance, emphasizing care vs. authority enhanced intergroup evaluations and increased willingness to engage in collective action in defense of immigrant rights (Argüello-Gutiérrez et al. 2024). Building on this, we hypothesized that activating care would decrease weight stigma, while activating purity would increase it.

## Transparency and openness

In all studies, we report how we determined our sample size, data exclusions, manipulations (if any), and measures. The data, analysis codes, and research materials are available at https://osf.io/bpheu. No data were collected after the data analysis began. Both Study 1 (https://osf.io/j4wmk) and Study 2 (https://osf.io/3jd6t) were pre-registered. Both studies complied with APA ethics principles. Given the observational and non-invasive nature of the online study, formal approval from Universidad Nacional de Educación a Distancia (UNED) institutional review board was not required.

## Study 1

Study 1 aimed to investigate the relationship between moral foundations, the moralization of obesity, and various measures of weight stigma, including all anti-fat attitudes subscales, and discrimination. Additionally, we explored how moral foundations relate to positive moral stereotypes and collective action. All analyses controlled for other influential factors, such as Body Mass Index (BMI) and self-perceived weight status.^2^

### Method

#### Participants and procedure

We used G*Power (Faul et al. 2017) to compute sample size for a small-medium effect in a multiple linear regression (two predictors, 80% power, *α* = .05; *f*^2^ = .04). The analysis revealed a minimum required sample size of *N* = 244. Three hundred and forty-seven Psychology students from a Spanish distance learning university participated in an online survey distributed via email. Students at this university exhibit greater sociodemographic diversity compared to those at traditional in-person universities (Sánchez-Elvira Paniagua et al. 2006). The age range of these students is broad, with a notable proportion being older adults (average age around 36 years, with a standard deviation of roughly 12 years). Additionally, the student body is dispersed throughout Spain, residing in both urban and rural areas.

The final sample, after excluding 96 participants who failed attention check (‘please select “*strongly agree*” to show you are paying attention to this question’), did not accept informed consent or quit the questionnaire before completing it, consisted of 251 individuals (189 women; *M_age_* = 40.10, *SD_age_* = 11.12; and 65.3% reporting an average or higher monthly income).

We informed participants that the research involved answering an anonymous questionnaire about morality and social perception. Once they agreed to participate, they first responded to sociodemographic questions, then completed the following scales.

#### Measures

Unless otherwise specified, scales ranged from 0 = *strongly disagree* to 6 = *strongly agree*.

##### Moral foundations

We assessed moral foundations using the 30-item version of the Moral Foundations Questionnaire (Graham et al. 2011, adapted to Spanish by Gudiño Paredes & Fernández Cárdenas 2015), which consists of two 15-item parts. The first part measures explicit thoughts about what is morally relevant (0 = *not at all relevant* to 6 = *extremely relevant*). The second part assesses the actual use of a moral foundation in judgement, with items rated on a scale from 0 = *strongly disagree* to 6 = *strongly agree*. The Cronbach’s alphas of our study were .67, .72, .65, .72, and .69 for care, fairness, loyalty, authority, and purity, respectively. The composite of individualizing foundations was calculated by averaging the Harm and Fairness subscales (*r*[249] = .66), while the composite for binding foundations was obtained by averaging the Ingroup, Authority, and Purity subscales (with correlations between these subscales ranging from .59 to .68). Therefore, each subscale score was calculated by averaging its corresponding items, and the composite scores for individualizing and binding foundations were computed by averaging their respective subscales. Exploratory factor analyses conducted on this scale yielded seven factors with eigenvalues greater than one. If a five-factor solution is forced, the five factors explain 52.1% of the variance (full factor loadings are available in the Supplementary Materials).

##### Moralization of obesity

We measured the extent to which participants morally disapprove obesity using five items used by Ringel and Ditto (2019): 1) gaining an excessive amount of weight is disrespectful to one’s body; 2) thinness is a moral virtue; 3) obesity is a moral failing; 4) obesity is a sign of personal weakness; 5) if a person is capable of being thin, they should be thin. Importantly, the original scale was designed to measure general moral disapproval of obesity and the type of absolutist thinking characteristic of moral attitudes. However, the three items that specifically assess absolutist thinking were not used in the present study, as they were beyond its objectives. Cronbach’s alpha for this scale was .81. Exploratory factor analyses conducted on the five items yielded a single factor (full factor loadings are available in the Supplementary Materials).

##### Moral stereotypes

To assess it, we asked participants to indicate to what extent they thought that the following traits described individuals with obesity: 1) honest; 2) fair; 3) sincere; 4) trustworthy; 5) morally committed; 6) generous; 7) with deep moral values; 8) ethically responsible. Participants rated each trait on a scale from 0 = *not at all* to 6 = *extremely*. Cronbach’s alpha for this scale was .94. Exploratory factor analyses conducted on the eight items yielded a single factor (full factor loadings are available in the Supplementary Materials). The first three items were drawn from Leach et al. (2007), who conceptualize morality as a central component of group impression formation. The remaining items have been used in previous research examining moral perceptions of ideological outgroups (Catena-Fernández & Fernández 2025). All items reflect morally positive traits; therefore, lower scores indicate a tendency to deny moral worth, which has been theorized as one-way stigmatized groups are devalued in moral terms (Brambilla et al. 2021).

##### Anti-fat attitudes

To assess the attitudes toward people with obesity we used the 13-item anti-fat attitudes (Crandall 1994, adapted to Spanish by Magallares and Morales 2014) scale. Seven items measured dislike of people with obesity (e.g., ‘I don’t have many friends that are fat,’ α = .83), three items measured fear of fat (e.g., ‘I worry about becoming fat,’ α = .80), and three items measured beliefs about willpower (e.g., ‘Some people are fat because they have no willpower,’ α = .83). We added three extra items related to the disgust subscale (e.g., ‘people with obesity disgust me,’ α = .72) and taken from Quinn and Crocker’s (1999) modification of the AFA scale. Exploratory factor analyses conducted on the sixteen items yielded four factors with eigenvalues greater than one. Together, the four factors explained 68.8% of the variance (full factor loadings are available in the Supplementary Materials).

##### Discrimination

We used two items adapted from Ringel and Ditto (2019) to assess discrimination against individuals with obesity: 1) People with obesity should have to pay for two seats on an airplane; 2) People with obesity should have to pay more for health insurance than thin people. Both items were significantly correlated (*r*[251] = .63, *p* < .001), so for the analysis we used the average score of both items.

##### Willingness to Participate in Collective Action for People with Obesity Rights

We assessed collective action by asking participants to what extent they were willing to engage in four actions on a scale adapted from Duncan (1999) ranging from 0 = *not willing at all* to 6 = *totally willing*: 1) Sign a petition in favor of the rights of people with obesity; 2) Donate money to defend the rights of people with obesity; 3) Actively participate in an organization that defends the rights of people with obesity; 4) Attending demonstrations to demand the rights of people with obesity. Cronbach’s alpha for this scale was .90. Exploratory factor analyses conducted on the five items yielded a single factor (full factor loadings are available in the Supplementary Materials).

##### Additional measures

We asked participants to provide their height and weight to calculate BMI. Based on standard classification criteria (BMI ≥ 30), 19 participants (7.6%) were categorized as obese. Participants also indicated their self-perceived weight on a scale from 1 to 4 (thin, average, overweight, obese). A total of 68 participants (14.5%) described themselves as either overweight or obese. Finally, we assessed political ideology on a scale ranging from 1 = *very left-winger* to 7 = *very right-winger*.

### Results

#### Correlations

Table 1 shows the descriptive statistics and correlations between the variables. Participants showed greater endorsement of individualizing foundations than binding foundations.

**Table 1:** Descriptive statistics and correlations among variables, Study 1.


VARIABLE	CORRELATIONS													DESCRIPTIVE STATISTICS

	1	2	3	4	5	6	7	8	9	10	11	12	13	*MEAN (SD)*

1. Individualizing	—													4.76 (0.73)

2. Binding	–.14*	—												2.74 (0.84)

3. Moralization of obesity	–.39***	.35***	—											1.32 (1.14)

4. Moral stereotypes	.09	.08	.01	—										2.57 (1.29)

5. Dislike	–.34***	.20**	.64***	–.05	—									0.74 (0.93)

6. Fear of fat	–15.*	.03	.38***	.03	.34***	—								2.08 (1.43)

7. Willpower	–.33***	.27***	.63***	.00	.54***	.28***	—							2.06 (1.55)

8. Disgust	–.34***	.15*	.50**	–.05	.69***	.29***	.48***	—						0.47 (0.95)

9. Discrimination	–.37*	.23***	.44***	–.05	.36***	.19**	.46***	.43***	—					0.91 (1.35)

10. Collective action	.37***	–.09	–.41***	.18**	–.36***	–.20**	–.50***	.37***	–.41***	—				2.78 (1.75)

11. Political ideology	–.39***	.50***	.43***	.03	.24***	.11	.32***	.21***	.35***	–.25***	—			3.04 (1.41)

12. BMI	–.05	.17*	.04	.13*	–.11	.14*	–.00	–.03	.01	.03	.03	—		24.16 (3.87)

13. Self-Perceived weight	.01	.04	–.03	.14*	–.19**	.18**	–.08	–.10	–.08	.04	–.09	.73***	—	2.16 (0.66)


Note: ***p < .001, **p < .01, *p < .05. Scales ranged from 0 to 6 except for political ideology (from 1 = very left-wing to 7 = very right-wing), and self-perceived weight (from 1 = thin to 4 = obese). BMI was calculated using the formula BMI = kg/m^2^.

As expected, endorsement of individualizing foundations was negatively associated with the moralization of obesity, all anti-fat attitudes and discrimination, and positively associated with collective action. Moral stereotypes were not significantly correlated with individualizing foundations. Also as expected, endorsement of binding foundations was positively associated with the moralization of obesity, dislike, willpower, disgust, and discrimination. Moral stereotypes, fear of fat, and collective action were not significantly correlated with binding foundations. Finally, the moralization of obesity was positively associated with all anti-fat attitudes and discrimination, and negatively correlated with collective action.

#### Regression analyses

We ran eight hierarchical linear regressions analyses to examine whether individualizing and binding foundations predicted each of the following variables: the moralization of obesity and moral stereotypes (see Table 2), dislike and fear of fat (see Table 3), willpower and disgust (see Table 4), as well as discrimination and support for collective action (see Table 5). In each regression, we controlled the influence of BMI and self-perceived weight. Specifically, BMI and self-perceived weight were included as predictors in Step 1, followed by individualizing and binding foundations in Step 2.

**Table 2:** Hierarchical regression for moralization of obesity and moral stereotypes, Study 1.


	MORALIZATION OF OBESITY					MORAL STEREOTYPES				
	
	*B*	*SE*	β	*t*	*p*	*B*	*SE*	β	*t*	*p*

(Constant)	.90	.47		1.91	.057	1.66	.53		3.13	.002

BMI	.03	.03	.12	1.27	.206	.02	.03	.06	.68	.495

Self-perceived weight	–.19	.16	–.11	–1.18	.239	.19	.18	.10	1.10	.274

	*F*(2, 248) = 0.88, *p* = .417 R^2^_adjusted_ = –.001					*F*(2, 248) = 2.84, *p* = .060 R^2^_adjusted_ = .022				

(Constant)	2.93	.63		4.66	<.001	.53	.80		.66	.509

BMI	–.01	.02	–.02	–.21	.832	.02	.03	.05	.50	.618

Self-perceived weight	–.04	.14	–.02	–.25	.803	.21	.18	.11	1.17	.243

Individualizing foundations	–.54	.09	–.35	–6.13	<.001	.18	.11	.10	1.62	.106

Binding foundations	.41	.08	.31	5.34	<.001	.13	.10	.09	1.35	.179

	*F*(4, 246) = 19.40, *p* < .001 Δ*F*(2, 246) = 37.66, Δ*R*^2^ = .233, *p* < .001					*F*(4, 246) = 2.42, *p* = .049 *F*(2, 246) = 1.98, Δ*R*^2^ = .016, *p* = .140				


Note: Each dependent variable was analyzed in a separate regression model. Variables are grouped in the table for presentation purposes only.

**Table 3:** Hierarchical regression for dislike and fear of fat, Study 1.


	DISLIKE					FEAR OF FAT				
	
	*B*	*SE*	β	*t*	*p*	*B*	*SE*	β	*t*	*p*

(Constant)	1.11	.38		2.91	.004	1.10	.59		1.86	.064

BMI	.01	.02	.06	.61	.542	.01	.03	.03	.33	.746

Self-perceived weight	–.32	.13	–.22	–2.50	.013	.34	.20	.15	1.71	.088

	*F*(2, 248) = 4.58, *p* = .011 R^2^_adjusted_ = .028					*F*(2, 248) = 3.97, *p* = .020 R^2^_adjusted_ = .023				

(Constant)	2.85	.54		5.30	<.001	2.58	.89		2.90	.004

BMI	–.01	.02	–.03	–.39	.697	.00	.03	.01	.13	.895

Self-perceived weight	–.23	.12	–.16	–1.96	.052	.37	.20	.17	1.86	.064

Individualizing foundations	–.41	.08	–.32	–5.43	<.001	–.29	.12	–.15	–2.39	.018

Binding foundations	.19	.07	.17	2.87	.004	.00	.11	.00	.03	,977

	*F*(4, 246) = 13.21, *p* < .001 Δ*F*(2, 246) = 21.10, Δ*R*^2^ = .141, *p* < .001					*F*(4, 246) = 3.47, *p* = .009 Δ*F*(2, 246) = 2.91, Δ*R*^2^ = .022, *p* = .057				


Note: Each dependent variable was analyzed in a separate regression model. Variables are grouped in the table for presentation purposes only.

**Table 4:** Hierarchical regression for willpower and disgust, Study 1.


	WILLPOWER					DISGUST				
	
	*B*	*SE*	β	*t*	*p*	*B*	*SE*	β	*t*	*p*

(Constant)	1.77	.64		2.76	.006	.45	.39		1.15	.249

BMI	.05	.04	.12	1.31	.192	.02	.02	.09	1.01	.312

Self-perceived weight	–.39	.21	–.17	–1.85	.066	–.24	.13	–.17	–1.86	.064

	*F*(2, 248) = 1.71, *p* = .184 R^2^_adjusted_ = .006					*F*(2, 248) = 1.83, *p* = .162 R^2^_adjusted_ = .007				

(Constant)	4.20	.90		4.69	<.001	2.38	.56		4.26	<.001

BMI	.01	.03	.01	.17	.868	.01	.02	.02	.26	.796

Self-perceived weight	–.23	.20	–.10	–1.16	.246	–.17	.12	–.12	–1.39	.165

Individualizing foundations	–.62	.12	–.29	–4.98	<.001	–.42	.08	–.33	–5.43	<.001

Binding foundations	.43	.11	.23	3.89	<.001	.12	.07	.11	1.77	.077

	*F*(4, 246) = 12.35, *p* < .001 Δ*F*(2, 246) = 22.70, Δ*R*^2^ = .154, *p* < .001					*F*(4, 246) = 9.92, *p* < .001 Δ*F*(2, 246) = 17.76, Δ*R*^2^ = .124, *p* < .001				


Note: Each dependent variable was analyzed in a separate regression model. Variables are grouped in the table for presentation purposes only.

**Table 5:** Hierarchical regression for discrimination and collective action, Study 1.


	DISCRIMINATION					COLLECTIVE ACTION				
	
	*B*	*SE*	β	*t*	*p*	*B*	*SE*	β	*t*	*p*

(Constant)	.49	.56		.88	.380	2.47	.73		3.39	<.001

BMI	.05	.03	.15	1.64	.102	.01	.04	.01	.16	.874

Self-perceived weight	–.39	.19	–.19	–2.07	.039	.07	.24	.03	.27	.785

	*F*(2, 248) = 2.17, *p* = .116 R^2^_adjusted_ = .009					*F*(2, 248) = .17, *p* = .846 R^2^_adjusted_ = –.007				

(Constant)	3.24	.78		4.17	<.001	–1.74	1.03		–1.68	.094

BMI	.02	.03	.06	.65	.518	.03	.04	.07	.82	.416

Self-perceived weight	–.25	.17	–.12	–1.48	.141	–.05	.23	–.02	–.20	.844

Individualizing foundations	–.64	.11	–.35	–5.91	<.001	.87	.14	.36	6.06	<.001

Binding foundations	.29	.10	.18	3.03	.003	–.11	.13	–.06	–.88	,381

	*F*(4, 246) = 13.62, *p* < .001 Δ*F*(2, 246) = 24.65, Δ*R*^2^ = .164, *p* < .001					*F*(4, 246) = 9.94, *p* < .001 Δ*F*(2, 246) = 19.69, Δ*R*^2^ = .138, *p* < .001				


Note: Each dependent variable was analyzed in a separate regression model. Variables are grouped in the table for presentation purposes only.

Importantly, each regression analysis was conducted independently, with one dependent variable analyzed per model. The dependent variables are presented in pairs in the tables for convenience, but they were not included in the same regression model.

As shown in Table 2, moral foundations significantly predict the moralization of obesity, but not moral stereotypes. As expected, individualizing foundations were negatively associated with the moralization of obesity (*B* = –.536, *SE* = .087, *t* = –6.13, *p* < .001), whereas binding foundations were positively associated with the moralization of obesity (*B* = .413, *SE* = .077, *t* = 5.33, *p* < .001). Contrary to our expectations, the relationship between both individualizing and binding foundations with moral stereotypes was non-significant (*B* = .180, *SE* = .111, *t* = 1.62, *p* = .106; and *B* = .133, *SE* = .099, *t* = 1.35, *p* = .179, respectively).

Table 3 shows that moral foundations significantly predict dislike, but only individualizing foundations predict fear of fat. As expected, individualizing foundations were negatively associated with dislike (*B* = –.406, *SE* = .075, *t* = –5.43, *p* < .001), whereas binding foundations were positively associated with dislike (*B* = .190, *SE* = .066, *t* = 2.87, *p* = .004). Also as expected, individualizing foundations were negatively associated with fear of fat (*B* = –.294, *SE* = .123, *t* = –2.39, *p* = .018). Contrary to our expectations, the relationship between binding foundations and fear of fat was non-significant (*B* = .003, *SE* = .109, *t* = 0.03, *p* = .977).

As indicated in Table 4, moral foundations significantly predict willpower, but only individualizing foundations predict disgust. As expected, individualizing foundations were negatively associated with willpower (*B* = –.620, *SE* = .124, *t* = –4.98, *p* < .001), whereas binding foundations were positively associated with willpower (*B* = .428, *SE* = .110, *t* = 3.86, *p* < .001). Also as expected, individualizing foundations were negatively associated with disgust (*B* = –.420, *SE* = .077, *t* = –5.43, *p* < .001). Binding foundations were positively associated with disgust, although the relationship did not reach statistical significance (*B* = .121, *SE* = .069, *t* = 1.77, *p* = .078).

Table 5 shows that moral foundations significantly predict discrimination, but only individualizing foundations predict support for collective action. As expected, individualizing foundations were negatively associated with discrimination (*B* = –.637, *SE* = .108, *t* = –5.91, *p* < .001), whereas binding foundations were positively associated with dislike of individuals with obesity (*B* = .289, *SE* = .095, *t* = 3.03, *p* = .003). Also as expected, individualizing foundations were positively associated with support for collective action (*B* = .870, *SE* = .144, *t* = 6.06, *p* < .001). Contrary to our expectations, the relationship between binding foundations and support for collective action was non-significant (*B* = –.112, *SE* = .127, *t* = –0.88, *p* = .381).

### Discussion

Our findings indicate that moral foundations were significant predictors of the moralization of obesity, anti-fat attitudes (particularly the dislike, willpower, and disgust subscales), and discrimination against individuals with obesity. Specifically, the endorsement of binding foundations was strongly associated with greater moral disapproval of obesity, increased dislike toward individuals with obesity, a stronger belief that weight is controllable, and a greater endorsement of discrimination against individuals with obesity. Binding foundations also tended to be associated with greater disgust towards individuals with obesity. This pattern may be explained by the perception that individuals with obesity violate norms of self-discipline or purity, which are central to binding foundations. This perception may evoke feelings of disgust or moral condemnation, as these individuals are seen as transgressing cultural expectations of health and self-control.

Conversely, the endorsement of individualizing foundations was strongly associated with less moral disapproval of obesity, more positive attitudes toward individuals with obesity, less endorsement of discrimination, and greater support for collective action advocating for the rights of people with obesity. This contrast underscores how individualizing foundations prioritize empathy and fairness over adherence to group norms or authority. For individuals with obesity, these foundations may counteract stigmatization by framing their experiences as issues of social injustice or systemic inequality rather than personal failings.

It is also worth noting that not all measures yielded strong associations with moral foundations. In particular, collective action and moral stereotypes showed weaker or non-significant patterns of results. The moral stereotype scale focused on the attribution of morally positive traits (e.g., honesty, sincerity) to individuals with obesity. While this approach offers insight into moral worth attribution, it may not fully capture the more commonly held negative stereotypes associated with obesity (e.g., laziness, lack of self-control). The collective action scale reflects downstream consequences of stigma rather than being a direct component of it, which could explain its weaker associations with moral values. These limitations informed us of our decision not to include these variables in study 2.

Additionally, we found a positive association between the moralization of obesity and all subscales of anti-fat attitudes and discrimination against people with obesity, replicating the findings of Ringel and Ditto (2019).

In summary, these results align with previous research indicating a strong relationship between endorsement of binding foundations and negative attitudes toward stigmatized groups (Argüello-Gutiérrez et al. 2024; Barnett et al. 2020; Kugler et al. 2014; Low & Wui 2016; Monroe & Plant 2019; Van de Vyver et al. 2016). However, given the limitations of correlational data in establishing causality, in Study 2, we experimentally tested the effects of framing individualizing versus binding moral arguments on the moralization of obesity, anti-fat attitudes, and discrimination against people with obesity.

## Study 2

Following the correlational results from Study 1, Study 2 explored the causal effect of moral framing on weight stigma. We manipulated moral framing to emphasize the societal benefits of advocating for care rather than purity. Given that moral stereotypes and collective action did not align with our expectations in Study 1, we excluded these variables from this study.

We expected that participants in the care (vs. purity) condition would exhibit reduced moralization of obesity, fewer anti-fat attitudes, and less discrimination against individuals with obesity. We also hypothesized that participants in the care (vs. purity) condition would report lower levels of anti-fat attitudes and discrimination against individuals with obesity via a stronger moral endorsement of obesity. Regarding the control condition, we expected it to yield intermediate levels of stigma compared to the other two conditions. Additionally, given previous findings on ideological differences in moral reasoning (Graham et al. 2013), we explored whether political ideology moderates these effects.^3^

### Method

#### Participants

We used G*Power (Faul et al. 2017) to conduct a priori power analysis (ANOVA: Three groups, fixed effects, omnibus, one-way; α = .05, and 80% power). Considering a small to medium effect size (Cohen’s *f* = .160, *η^2^* = .026), the analysis revealed a minimum required sample size of *N* = 381. We recruited 480 participants by using the procedure described in Study 1. Ninety-nine failed attention check (‘please select “*strongly agree*” to show you are paying attention to this question’), did not accept informed consent, or quit the questionnaire before completing it, leaving 381 participants for the analyses (68% women; *M_age_* = 41.25, *SD_age_* = 11.99; and 66.2% reporting an average or higher monthly income).

#### Procedure

We informed participants that the questionnaire dealt with the well-being and happiness of people in different cultures. Participants first answered the socio-demographic questions as in Study 1. Subsequently, they were randomly assigned to one of the three conditions (care vs. purity vs. control).

To manipulate this independent variable, we used the framework from Argüello-Gutiérrez et al. (2024). The goal of this manipulation was to momentarily activate care or purity moral concerns by presenting them as contributing to societal well-being. Specifically, in the care condition, participants learned that societies where people value compassion and show protective behaviors enjoy greater well-being and happiness. In the purity condition, participants learned that societies where people value the sacredness of life and display decent behaviors enjoy greater well-being and happiness. These manipulation texts were designed to highlight the societal relevance of each moral value and thus activate it as a salient lens for interpreting social issues.

Finally, participants in the control condition learned that societies where people value entertainment and engage in leisure activities enjoy greater well-being and happiness (The full texts used for each condition are available at https://osf.io/bpheu/). This control text was intended to be affectively positive but morally neutral. However, we acknowledge that some leisure activities—such as walking—could be interpreted as implicitly health-related, potentially introducing unintended associations with body weight. We return to this limitation in the discussion.

After the manipulation, participants proceeded to answer the items described below. Upon completion, we thanked them for their participation and explained the study’s goals.

#### Measures

Unless otherwise specified, scales ranged from 0 = *strongly disagree* to 6 = *strongly agree*.

We used the same items as in Study 2 to measure the moralization of obesity (α = .78), dislike (α = .81), fear of fat (α = .81), willpower (α = .77), disgust (α = .90), discrimination (α = .68), BMI (*M* = 24.41; *SD* = 4.13; 26 participants [6.8%] were categorized as obese), self-perceived weight (*M* = 1.21; *SD* = 0.71; 13 participants [3.4%] described themselves as either overweight or obese), and political ideology (*M* = 3.24; *SD* = 1.47). As in Study 1, full exploratory factor analysis is available in the Supplementary Materials.

To assess the perceived credibility of our manipulation, we used five items adapted from Argüello-Gutiérrez et al. (2024): ‘To what extent do you find the research we told you about at the beginning on the factors that influence the perception of well-being and happiness was.… 1) convincing; 2) plausible; 3) realistic; 4) credible; 5) possible.’ Cronbach’s alpha for this scale was .95. Participants responded on a scale from 0 = *not at all* to 6 = *very much*. Exploratory factor analyses conducted on the five items yielded a single factor (full factor loadings are available in the Supplementary Materials).

A one-way ANOVA on this measure with the condition as independent variable revealed a significant effect of the condition (*F*[2, 378] = 3.24, *p* = .040, *η^2^* = .017). Bonferroni post-hoc test indicated that participants in the control condition perceived the manipulation text as more credible than participants in the purity condition, although the effect comparison did not reach statistical significance (Mean Difference = 0.425, *p* = .056, *d* = 0.28, 95% CI [–0.0076, 0.8568]). The differences between the control and the care conditions, and between the care and purity conditions were nonsignificant (Mean Difference = 0.063, *p* = 1.00, *d* = 0.05, 95% CI [–0.3679, 0.4947], and Mean Difference = 0.361, *p* = .137, *d* = 0.25, 95% CI [–0.0718, 0.7943], respectively). Taken together, these findings confirmed that all three texts were rated as similarly credible.

### Results

First, we present the results of the one-way ANOVAs for all the dependent variables with the condition (control vs care vs purity) as independent variable, followed by Bonferroni post-hoc comparisons. Next, we present the results of the mediation models to test the hypothesized conditional indirect effects. Table 6 shows the zero-order correlations between all the variables.

**Table 6:** Correlations among variables, Study 2.


VARIABLE	1	2	3	4	5	6	7	8	9

1. Moralization of obesity	—								

2. Dislike	.49***	—							

3. Fear of fat	.34***	.35***	—						

4. Willpower	.63***	.41***	.26***	—					

5. Disgust	.48***	.70***	.34***	.34***	—				

6. Discrimination	.50***	.52***	.31***	.45***	.52***	—			

7. Political ideology	.34***	.26***	.01	.28***	.22***	.19***	—		

8. BMI	–.01.	–.10	.08	–.12*	–.04	–.10*	.12*	—	

9. Self-perceived weight	–.06	–.14**	.25***	–.15**	–.07	–.15**	–.02	.74***	—


Note: ***p < .001, **p < .01, *p < .05.

#### ANOVA

**Figure 1:**
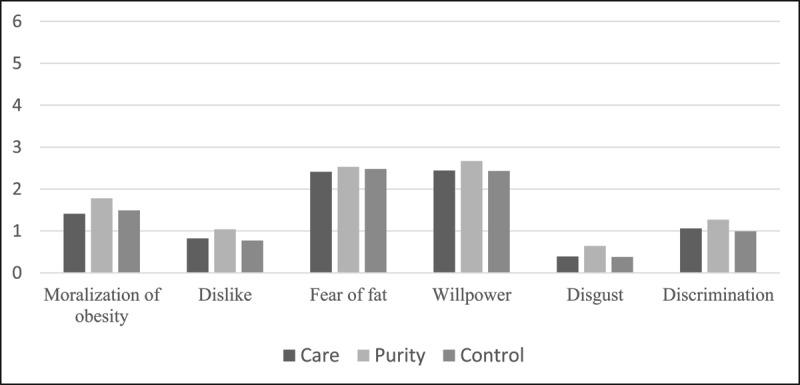
Descriptive statistics of all dependent variables across condition.

To test our hypothesis, we conducted a one-way ANOVA on all dependent variables and performed two planned contrasts.^4^ The first contrast compared participants in the care condition (coded –1) to those in the purity condition (coded +1), setting the control condition to 0. This tested our primary hypothesis that care foundation would reduce weight stigma compared to purity foundation. The second contrast compared the control condition (+2) to the average of the care and purity conditions (both coded –1), testing the hypothesis that the control condition would produce intermediate levels of stigma relative to the other two conditions. Table 7 shows the one-way ANOVA results for all dependent variables and descriptive statistics across conditions. Figure 1 shows a graphic representation of the means of all dependent variables across conditions.

**Table 7:** One-way ANOVA results for all dependent variables and descriptive statistics across conditions, Study 2.


VARIABLE	CONDITION						ANOVA		
	
	CARE		PURITY		CONTROL		*F*[2, 378]		
			
	*M*	*SD*	*M*	*SD*	*M*	*SD*	*F*	*p*	*η^2^*

Moralization of obesity	1.41	1.07	1.78	1.22	1.49	1.05	3.97	.020	.021

Dislike	0.82	0.85	1.04	0.96	0.77	0.70	3.64	.027	.019

Fear of fat	2.41	1.37	2.53	1.55	2.48	1.46	0.21	.808	.001

Willpower	2.44	1.33	2.67	1.44	2.43	1.32	1.20	.304	.006

Disgust	0.39	0.76	0.64	1.10	0.38	0.55	4.02	.019	.021

Discrimination	1.06	1.24	1.27	1.60	0.98	1.14	1.53	.219	.008

Manipulation Check	3.89	1.29	3.52	1.55	3.95	1.44	3.24	.040	.017


Note: Scales ranged from 0 to 6.

The results for moralization of obesity yielded a significant effect of the condition (*F*[2, 378] = 3.97, *p* = .020, *η^2^* = .021). The first planned contrast was significant, indicating that participants in the purity condition showed greater moralization of obesity than participants in the care condition, *t*(378) = 2.67, *p* = .008, *d* = 0.28, 95% CI [0.0988, 0.6505]). The second contrast (testing whether the control condition produced intermediate levels of stigma relative to the care and purity conditions) was nonsignificant, *t*(378) = 0.90, *p* = .367, *d* = 0.09, 95% CI [–0.2573, 0.6944].

The results for dislike yielded a significant effect of the condition (*F*[2, 378] = 3.64, *p* = .027, *η^2^* = .019). The first planned contrast was significant, indicating that participants in the purity condition perceived more dislike toward individuals with obesity than participants in the control condition, *t*(378) = 2.11, *p* = .035, *d* = 0.22, 95% CI [0.0154, 0.4329]. The second contrast (testing whether the control condition produced intermediate levels of stigma relative to the care and purity conditions) was nonsignificant, *t*(378) = 1.68, *p* = .093, *d* = 0.25, 95% CI [–0.0518, 0.6685].

The results for fear of fat and willpower yielded a nonsignificant effect of the condition (*F*[2, 378] = 0.21, *p* = .808, *η^2^* = .001, and *F*[2, 378] = 1.20, *p* = .304, *η^2^* = .006, respectively).

The results for disgust yielded a significant effect of the condition (*F*[2, 378] = 3.97, *p* = .020, *η^2^* = .021). The first planned contrast was significant, indicating that participants in the purity condition showed more disgust toward individuals with obesity than participants in the care condition, *t*(378) = 2.36, *p* = .019, *d* = 0.24, 95% CI [–0.0416, 0.4567]. The second contrast (testing whether the control condition produced intermediate levels of stigma relative to the care and purity conditions) was nonsignificant, *t*(378) = 1.57, *p* = .116, *d* = 0.16, 95% CI [–0.0715, 0.6446].

The results for discrimination yielded a nonsignificant effect of the condition (*F*[2, 378] = 1.53, *p* = .219, *η^2^* = .008).

#### Mediation models

We used Hayes’ (Model 4, 2017) PROCESS macro for IBM SPSS (Version 27, 2020) to test the indirect effects of the manipulation on dislike and disgust via moralization of obesity. Given that the status manipulation had three levels, we created two contrast-coded variables. The first contrast (C1) compared the care and purity conditions (care = –1, purity = +1, control = 0), testing our central hypothesis that care framing would reduce weight stigma compared to purity framing. The second contrast (C2) tested whether the control condition elicited intermediate levels of stigma relative to the other two (care = –1, purity = –1, control = +2). We therefore conducted four mediation models (i.e., two contrasts for two dependent variables). All analyses included BMI, self-perceived weight, and political ideology as covariates.

In the mediation analysis for dislike, the contrast C1 was significantly associated with moralization of obesity (*b* = .155, *SE* = .067, *p* = .020, 95% CI [0.0246, 0.2861]). Moralization of obesity, in turn, was positively associated with dislike (*b* = .338, *SE* = .036, *p* < .001, 95% CI [0.2670, 0.4081]). The indirect effect of C1 on dislike via moralization of obesity was significant (*b* = .053, Boot *SE* = .026, 95% CI [0.0075, 0.1081]), indicating that the care versus purity contrast affected dislike indirectly via moralization of obesity. The direct effect of C1 on dislike was nonsignificant (*b* = .039, *SE* = .047, *p* = .403, 95% CI [–0.0526, 0.1306]). The contrast C2 was not significantly associated with moralization of obesity (*b* = –.053, *SE* = .038, *p* = .165, 95% CI [–0.1289, 0.0221]). Moralization of obesity predicted dislike (*b* = .337, *SE* = .036, *p* < .001, 95% CI [0.2668, 0.4068]). The indirect effect of C2 on dislike was nonsignificant (*b* = –.018, Boot *SE* = .014, 95% CI [–0.0474, 0.0066]), and the direct effect was also nonsignificant (*b* = –.045, *SE* = .027, *p* = .093, 95% CI [–0.0970, 0.0075]).

In the mediation analysis for disgust, the contrast C1 was significantly associated with moralization of obesity (*b* = .155, *SE* = .067, *p* = .020, 95% CI [0.0246, 0.2861]). Moralization of obesity, in turn, was positively associated with disgust (*b* = .339, *SE* = .036, *p* < .001, 95% CI [0.2671, 0.4102]). The indirect effect of C1 on disgust via moralization of obesity was significant (*b* = .053, Boot *SE* = .027, 95% CI [0.0067, 0.1128]), indicating that the care versus purity contrast affected disgust indirectly via moralization of obesity. The direct effect of C1 on disgust was nonsignificant (*b* = .056, *SE* = .047, *p* = .236, 95% CI [–0.0368, 0.1490]). The contrast C2 was not significantly associated with moralization of obesity (*b* = –.053, *SE* = .038, *p* = .165, 95% CI [–0.1289, 0.0221]). Moralization of obesity predicted disgust (*b* = .340, *SE* = .036, *p* < .001, 95% CI [0.2690, 0.4113]). The indirect effect of C2 on disgust was nonsignificant (*b* = –.018, Boot *SE* = .014, 95% CI [–0.0495, 0.0067]), and the direct effect was also nonsignificant (*b* = –.039, *SE* = .027, *p* = .155, 95% CI [–0.0916, 0.0146]).

### Discussion

Study 2 suggests that emphasizing the foundation of purity in society can play an important role in weight stigma. Participants confronted with the social benefits of purity moralized obesity more and showed greater dislike and disgust toward people with obesity. Additionally, the observed conditional indirect effects provided correlational evidence about the key role that moralization of obesity may play on the weight stigma triggered by purity.

While these findings reinforce the results of Study 1 and suggest that binding foundations, particularly purity, can increase negative attitudes towards obesity, some limitations of the design should be acknowledged. First, the manipulation checks measured perceived plausibility and credibility of the moral framing texts but did not directly assess whether the care or purity values were activated. Future research should include a direct measure of moral value salience to confirm the psychological activation of these moral domains. Second, although the control condition was intended to provide a neutral comparison, the focus on leisure activities (e.g., going for a walk) might have inadvertently elicited health or body-related associations, potentially compromising their role as a truly neutral baseline condition. These issues should be addressed in future work aiming to test the causal role of moral framing in the weight stigma.

## General Discussion

The present research sought to explore the relationship between moral foundations, the moralization of obesity and weight stigma. Across two studies conducted in Spain, we examined how binding foundations and individualizing foundations differentially predict the moralization of obesity and weight stigma, and how manipulating the foundations of care and purity influences moral disapproval of obesity and anti-fat attitudes.

The findings of Study 1 supported the hypothesis that endorsement of binding foundations was positively associated with the moralization of obesity, anti-fat attitudes (particularly the dislike, willpower, and disgust subscales), and discrimination against individuals with obesity. Conversely, endorsement of individualizing foundations was negatively associated with the moralization of obesity, anti-fat attitudes, and discrimination against individuals with obesity, while also being positively associated with support for the rights of individuals with obesity.

Study 2 extended these findings by experimentally manipulating a moral framework that emphasized the societal benefits of prioritizing care versus purity. As predicted, participants exposed to the social benefits of purity showed greater moralization of obesity and higher levels of dislike and disgust toward individuals with obesity compared to those in the care and control conditions. Importantly, Study 2 also provided correlational evidence highlighting the key role that the moralization of obesity may play in weight stigma, particularly when triggered by purity-based moral framing.

Our research makes several key contributions to the literature on moral foundations and prejudice. First, it replicates the association between moral foundations and attitudes toward stigmatized groups observed in previous research (Argüello-Gutiérrez et al. 2024; Barnett et al. 2020; Koleva et al. 2012; Kugler et al. 2014; Low & Wui, 2016; Masicampo et al. 2014; Monroe & Plant 2019; Van de Vyver et al. 2016). By applying this framework to weight stigma, our research expands the relevance of moral foundations theory in understanding attitudes toward individuals with obesity.

Second, our experimental manipulation in Study 2 addresses a gap in literature, which has mainly relied on correlational methods to examine moral foundations and social attitudes toward stigmatized groups (e.g., Barnett et al. 2020; Kugler et al. 2014; Low & Wui 2016; Van de Vyver et al. 2016). By providing causal evidence for how purity and care influence weight stigma, our study responds to calls for experimental approaches to explore the effects of moral foundations on intergroup relations (Graham et al. 2013; Talaifar & Swann 2019).

Third, our findings provide correlational and experimental evidence on the relationship between moral foundations and weight stigma in Western Europe. Notably, most existing studies have been conducted in North America (e.g., Barnett et al. 2020; Kugler et al. 2014; Low & Wui 2016; Monroe & Plant 2019; Van de Vyver et al. 2016). By conducting this research in Spain, we contribute to a broader understanding of these dynamics in an underrepresented cultural context.

Fourth, this is the first study to apply MFT to weight stigma. While previous research has explored moral disgust and negative attitudes toward obesity (Clifford & Wendell 2016; Lieberman et al. 2012; Park et al. 2007; Vartanian 2010), no study has examined the causal link between purity and weight stigma. Our findings suggest that individuals exposed to purity-based moral framing may perceive obesity as a moral failing, leading to greater stigmatization. This insight fills an important gap and may inspire future research on the moral dimensions of weight stigma.

Importantly, while our findings support the role of moralization as a mechanism intensifying weight stigma, we acknowledge the possibility that moralization and stigma may be conceptually overlapping constructs. Some scholars argue that stigma itself often entails implicit moral condemnation (Yang et al. 2007), raising the question of whether moralization should be treated as a distinct antecedent or rather as a dimension of stigma. We adopted the former approach based on prior conceptual and empirical work (e.g., Ringel & Ditto 2019; Rozin 1999), which treats moralization as a specific process involving explicit ethical blame, thus allowing for the examination of its predictive role. Our data support this distinction, as moralization mediated the effects of moral foundations on stigmatizing attitudes. Nevertheless, future research should further explore the boundaries and interplay between these constructs.

The findings of this research also contribute to the literature on weight stigma in two ways. First, although previous studies have highlighted the strong relationship between moral disgust and negative attitudes toward individuals with obesity (e.g., Clifford & Wendell 2016; Lieberman et al. 2012; Park et al. 2007; Vartanian 2010), no prior research has examined the causal relationship between the moral foundation of purity and weight stigma. Our study fills this important gap and may pave the way for a new line of inquiry, suggesting that individuals exposed to the social benefits of purity may perceive obesity as a moral failing, thereby leading to increased stigmatization of people with obesity. We replicate the findings of Ringel and Ditto (2019), who demonstrated that the moralization of obesity predicts anti-fat attitudes and discrimination.

Our findings suggest that reducing weight stigma requires emphasizing individualizing moral values like empathy, compassion, and fairness in public health messaging to foster more inclusive attitudes. At the same time, addressing the negative impact of binding foundations, particularly purity, involves challenging the moralization of obesity by highlighting its genetic, environmental, and socio-economic influences, shifting the focus away from individual blame and moral judgment (Puhl 2022). Given that weight stigma is not only a social issue but also contributes to adverse physical and mental health outcomes (Major et al. 2017), our findings underscore the urgent need to address its moral roots.

While the present research provides valuable insights, it is not without limitations. One primary limitation is that the causal effects found in Study 2 remain unclear, though they establish a foundation for testing moral framing’s impact on weight stigma. Future longitudinal studies could examine whether changes in moral framing sustainably reduce stigma over time. Second, Study 2 focused only on care and purity, while Study 1 examined all moral foundations. Future studies should manipulate authority/respect and fairness/reciprocity, which may play a stronger role in the moralization of obesity and weight stigma. Third, conducting the study in Spain may limit its cultural generalizability. Further research should explore these relationships in diverse cultural contexts. Finally, while we tested mediation models, the cross-sectional design prevents inferring causal chains. Despite this, our findings provide a meaningful basis for future research.

In conclusion, this research enhances understanding of the moral roots of weight stigma and highlights the need to consider moral values in efforts to reduce it. Promoting individualizing foundations while addressing the moralization tied to binding foundations can help public health initiatives foster a more inclusive and supportive environment for individuals with obesity.

## Additional File

The additional file for this article can be found as follows:

10.5334/irsp.1068.s1 Supplementary Material. Studies 1 and 2.

## Footnotes

Atari et al. (2023) presented evidence for a factor structure incorporating a sixth dimension. Nevertheless, moral foundation questionnaires that utilize a five-dimension model continue to be effective, showing sufficient measurement invariance (Nilsson 2022).

Supplementary Materials include additional analyses controlling political ideology.

Political ideology was preregistered as a potential moderator and is included here as part of secondary analyses. Full results are reported in the Supplementary Materials.

Thanks to a pertinent comment from the reviewer, we decided during the review process to conduct planned contrasts instead of the Bonferroni post-hoc comparisons, which are the ones we pre-registered. Planned contrasts are theoretically justified and more statistically powerful for testing our hypotheses.

## References

[1] Abele, A. E., Ellemers, N., Fiske, S. T., Koch, A., & Yzerbyt, V. (2021). Navigating the social world: Toward an integrated framework for evaluating self, individuals, and groups. Psychological Review, 128, 290–314. 10.1037/rev0000262 32940512

[2] Argüello-Gutiérrez, C., López-Rodríguez, L., & Vázquez, A. (2024). The effect of moral foundations on intergroup relations: The salience of fairness promotes the acceptance of minority groups. Social Psychological and Personality Science, 15, 93–105. 10.1177/19485506231162161

[3] Atari, M., Haidt, J., Graham, J., Koleva, S., Stevens, S. T., & Dehghani, M. (2023). Morality beyond the WEIRD: How the nomological network of morality varies across cultures. Journal of Personality and Social Psychology, 125, 1157–1188. 10.1037/pspp0000470 37589704

[4] Barnett, M. D., Maciel, I. V., & Sligar, K. B. (2020). Moral foundations, sexual prejudice, and outness among sexual minorities. Sexuality & Culture, 24, 1387–1396. 10.1007/s12119-019-09689-1

[5] Brambilla, M., Sacchi, S., Rusconi, P., & Goodwin, G. P. (2021). The primacy of morality in impression development: Theory, research, and future directions. In Advances in experimental social psychology (Vol. 64, pp. 187–262). Academic Press. 10.1016/bs.aesp.2021.03.001

[6] Carvalho, K., Peretz-Lange, R., & Muentener, P. (2021). Causal explanations for weight influence children’s social preferences: Biological-essentialist explanations reduce, and behavioral explanations promote, preferences for thin friends. Child Development, 92, 682–690. 10.1111/cdev.13519 33521936

[7] Catena-Fernández, C., & Fernández, S. (2025). Moral perceptions about environmentalism across ideological groups: Negative moral stereotypes threaten rightists’ moral self and trigger polarization. Group Processes & Intergroup Relations. 10.1177/13684302251330990

[8] Clifford, S., & Wendell, D. G. (2016). How disgust influences health purity attitudes. Political Behavior, 38, 155–178. 10.1007/s11109-015-9310-z

[9] Crandall, C. S. (1994). Prejudice against fat people: Ideology and self-Interest. Journal of Personality and Social Psychology, 66, 882–894. 10.1037/0022-3514.66.5.882 8014833

[10] Day, M. V., Fiske, S. T., Downing, E. L., & Trail, T. E. (2014). Shifting liberal and conservative attitudes using moral foundations theory. Personality and Social Psychology Bulletin, 40, 1559–1573. 10.1177/0146167214551152 25286912 PMC4858184

[11] Duncan, L. E. (1999). Motivation for collective action: Group consciousness as mediator of personality, life experiences, and women’s rights activism. Political Psychology, 20, 611–635. 10.1111/0162-895X.00159

[12] Elison, Z. M., & Çiftçi, A. (2015). Digesting antifat attitudes: Locus of control and social dominance orientation. Translational Issues in Psychological Science, 1, 262–270. 10.1037/tps0000029

[13] Faul, F., Erdfelder, E., Buchner, A., & Lang, A. G. (2017). G*power 3.1 manual. Universitat Bonn.

[14] Feijoo, L., Rey-Brandariz, J., Guerra-Tort, C., Candal-Pedreira, C., Santiago-Pérez, M. I., Ruano-Ravina, A., & Pérez-Ríos, M. (2024). Prevalence of obesity in Spain and its autonomous communities, 1987–2020. Revista Española de Cardiología (English Edition), 77(10), 809–818. 10.1016/j.rec.2023.12.018 38490640

[15] Gaspar, M. C. D. M. P., de Morais Sato, P., & Scagliusi, F. B. (2022). Under the ‘weight’ of norms: Social representations of overweight and obesity among Brazilian, French and Spanish dietitians and laywomen. Social Science & Medicine, 298, 114861. 10.1016/j.socscimed.2022.114861 35228094

[16] Gracia-Arnaiz, M. (2013). Thou shalt not get fat: medical representations and self-images of obesity in a Mediterranean society. Health, 5, 1180–1189. 10.4236/health.2013.57159

[17] Graham, J., Haidt, J., Koleva, S., Motyl, M., Iyer, R., Wojcik, S. P., & Ditto, P. H. (2013). Moral foundations theory: The pragmatic validity of moral pluralism. Advances in Experimental Social Psychology, 47, 55–130. 10.1016/B978-0-12-407236-7.00002-4

[18] Graham, J., Haidt, J., & Nosek, B. A. (2009). Liberals and conservatives rely on different sets of moral foundations. Journal of Personality and Social Psychology, 96, 1029–1046. 10.1037/a0015141 19379034

[19] Graham, J., Nosek, B. A., Haidt, J., Iyer, R., Spassena, K., & Ditto, P. H. (2011). Moral foundations questionnaire (MFQ). [Database record]. Retrieved from APA PsycTests. 10.1037/t05651-000

[20] Gudiño Paredes, S., & Fernández Cárdenas, J. M. (2015). Propiedades psicométricas del Cuestionario de Fundamentos Morales en alumnos de bachillerato: Un estudio exploratorio. Enseñanza e Investigación en Psicología, 20, 130–139. https://www.redalyc.org/articulo.oa?id=29242799003

[21] Haidt, J. (2012). The righteous mind: Why good people are divided by politics and religion. Vintage.

[22] Horberg, E. J., Oveis, C., Keltner, D., & Cohen, A. B. (2009). Disgust and the moralization of purity. Journal of Personality and Social Psychology, 97, 963–976. 10.1037/a0017423 19968413

[23] IBM Corp. (2020). IBM SPSS Statistics for Windows (Version 27.0.) [Computer software].

[24] Koleva, S. P., Graham, J., Iyer, R., Ditto, P. H., & Haidt, J. (2012). Tracing the threads: How five moral concerns (especially Purity) help explain culture war attitudes. Journal of Research in Personality, 46, 184–194. 10.1016/j.jrp.2012.01.006

[25] Kugler, M., Jost, J. T., & Noorbaloochi, S. (2014). Another look at moral foundations theory: Do authoritarianism and social dominance orientation explain liberal-conservative differences in “moral” intuitions? Social Justice Research, 27, 413–431. 10.1007/s11211-014-0223-5

[26] Leach, C. W., Ellemers, N., & Barreto, M. (2007). Group virtue: The importance of morality (vs. competence and sociability) in the positive evaluation of in-groups. Journal of Personality and Social Psychology, 93, 234–249. 10.1037/0022-3514.93.2.234 17645397

[27] Lieberman, D. L., Tybur, J. M., & Latner, J. D. (2012). Disgust sensitivity, obesity stigma, and gender: Contamination psychology predicts weight bias for women, not men. Obesity, 20, 1803–1814. 10.1038/oby.2011.247 21836644

[28] Low, M., & Wui, M. G. L. (2016). Moral foundations and attitudes towards the poor. Current Psychology, 35, 650–656. 10.1007/s12144-015-9333-y

[29] Magallares, A. (2014). Right wing autoritharism, social dominance orientation, controllability of the weight and their relationship with antifat attitudes. Universitas Psychologica, 13, 15–23. 10.11144/Javeriana.UPSY13-2.rwas

[30] Magallares, A., & Morales, J. F. (2014). Spanish adaptation of the Antifat Attitudes Scale/Adaptación al castellano de la Escala de Actitud Antiobesos. International Journal of Social Psychology, 29, 563–588. 10.1080/02134748.2014.972707

[31] Magallares, A., Recio, P., Jáuregui-Lobera, I., de Valle, P. B., Irles, J. A., & Hymowitz, G. (2024). Factor structure and measurement invariance of the Weight-Related Abuse Questionnaire (WRAQ). Eating Behaviors, 52, 101827. 10.1016/j.eatbeh.2023.101827 38007887

[32] Major, B., Tomiyama, J., & Hunger, J. M. (2017). The negative and bidirectional effects of weight stigma on health. In B. Major, J. F. Dovidio & B. G. Link (Eds.), Oxford library of psychology. The Oxford handbook of stigma, discrimination, and health (pp. 499–519). Oxford University Press. 10.1093/oxfordhb/9780190243470.013.27

[33] Masicampo, E. J., Barth, M., & Ambady, N. (2014). Group-based discrimination in judgments of moral purity-related behaviors: Experimental and archival evidence. Journal of Experimental Psychology: General, 143, 2135–2152. 10.1037/a0037831 25199041

[34] Milesi, P. (2017). Moral foundations and voting intention in Italy. Europe’s Journal of Psychology, 13, 667–687. 10.5964/ejop.v13i4.1391 PMC5763456 29358981

[35] Ministerio de Sanidad. (2023). Informe anual del Sistema Nacional de Salud 2022. https://www.sanidad.gob.es/estadEstudios/estadisticas/sisInfSanSNS/tablasEstadisticas/InfAnualSNS2023/INFORME_ANUAL_2023.pdf

[36] Monroe, A. E., & Plant, E. A. (2019). The dark side of morality: Prioritizing sanctity over care motivates denial of mind and prejudice toward sexual outgroups. Journal of Experimental Psychology: General, 148, 342–360. 10.1037/xge0000537 30570329

[37] Mooijman, M., Meindl, P., Oyserman, D., Monterosso, J., Dehghani, M., Doris, J. M., & Graham, J. (2018). Resisting temptation for the good of the group: Binding moral values and the moralization of self-control. Journal of Personality and Social Psychology, 115, 585–599. 10.1037/pspp0000149 28604018

[38] Nilsson, A. (2022). Measurement invariance of moral foundations across population strata. Journal of Personality Assessment, 105, 163–173. 10.1080/00223891.2022.2074853 35583925

[39] O’Brien, K. S., Latner, J. D., Ebneter, D., & Hunter, J. A. (2013). Obesity discrimination: The role of physical appearance, personal ideology, and anti-fat prejudice. International Journal of Obesity, 37, 455–460. 10.1038/ijo.2012.52 22531085

[40] Park, J. H., Schaller, M., & Crandall, C. S. (2007). Pathogen-avoidance mechanisms and the stigmatization of obese people. Evolution and Human Behavior, 28, 410–414. 10.1016/j.evolhumbehav.2007.05.008

[41] Peretz-Lange, R., Carvalho, K., & Muentener, P. (2024). Children’s essentialist beliefs about weight. Journal of Cognition and Development, 25, 1–26. 10.1080/15248372.2023.2237228

[42] Puhl, R. M. (2022). Weight stigma, policy initiatives, and harnessing social media to elevate activism. Body Image, 40, 131–137. 10.1016/j.bodyim.2021.12.008 34953387

[43] Puhl, R. M., Lessard, L. M., Pearl, R. L., Himmelstein, M. S., & Foster, G. D. (2021). International comparisons of weight stigma: Addressing a void in the field. International Journal of Obesity, 45, 1976–1985. 10.1038/s41366-021-00860-z 34059785

[44] Quinn, D. M., & Crocker, J. (1999). When ideology hurts: Effects of belief in the protestant ethic and feeling overweight on the psychological well-being of women. Journal of Personality and Social Psychology, 77, 402–414. 10.1037/0022-3514.77.2.402 10474214

[45] Ringel, M. M., & Ditto, P. H. (2019). The moralization of obesity. Social Science & Medicine, 237, 112399. 10.1016/j.socscimed.2019.112399 31377501

[46] Rozin, P. (1999). The process of moralization. Psychological Science, 10, 218–221. 10.1111/1467-9280.00139

[47] Rubino, F., Puhl, R. M., Cummings, D. E., Eckel, R. H., Ryan, D. H., Mechanick, J. I., … & Dixon, J. B. (2020). Joint international consensus statement for ending stigma of obesity. Nature Medicine, 26, 485–497. 10.1038/s41591-020-0803-x PMC7154011 32127716

[48] Sánchez-Elvira Paniagua, Á., Viedma Rojas, A., Gómez Garrido, M., Santiago Alba, C. D., & Ortí Mata, M. (2006). Actitudes y valores hacia la ciencia y la tecnología en los alumnos del curso de acceso para mayores de 25 años de la UNED [Attitudes and values towards science and technology in the students of the access course for over 25 years of the UNED]. Fundación Española para la Ciencia y la Tecnología. http://e-spacio.uned.es/fez/view/bibliuned:500796

[49] Talaifar, S., & Swann, W. B. (2019). Deep alignment with country shrinks the moral gap between conservatives and liberals. Political Psychology, 40, 657–675. 10.1111/pops.12534

[50] Van de Vyver, J., Houston, D. M., Abrams, D., & Vasiljevic, M. (2016). Boosting belligerence: How the July 7, 2005, London bombings affected liberals’ moral foundations and prejudice. Psychological Science, 27, 169–177. 10.1177/0956797615615584 26674127 PMC4750069

[51] Vartanian, L. R. (2010). Disgust and perceived control in attitudes toward obese people. International Journal of Obesity, 34, 1302–1307. 10.1002/oby.20721 20195287

[52] Wagemans, F., Brandt, M. J., & Zeelenberg, M. (2018). Disgust sensitivity is primarily associated with purity-based moral judgments. Emotion, 18, 277–289. 10.1037/emo0000359 28872334

[53] Winterich, K. P., Zhang, Y., & Mittal, V. (2012). How political identity and charity positioning increase donations: Insights from moral foundations theory. International Journal of Research in Marketing, 29, 346–354. 10.1016/j.ijresmar.2012.05.002

[54] Woodhouse, R. (2008). Obesity in art–a brief overview. Obesity and Metabolism, 36, 271–286. 10.1159/000115370 18230908

[55] Yang, L. H., Kleinman, A., Link, B. G., Phelan, J. C., Lee, S., & Good, B. (2007). Culture and stigma: Adding moral experience to stigma theory. Social Science & Medicine, 64, 1524–1535. 10.1016/j.socscimed.2006.11.013 17188411

