# Supplementary material for: Moral Foundations and Obesity: The Role of Binding vs. Individualizing Foundations in Shaping Weight Stigma
Source: Int Rev Soc Psychol. 2025 Jul 18;38:11. doi: 10.5334/irsp.1068 (PMC12372685; doi:10.5334/irsp.1068)
Supplement: Supplementary Material. — Studies 1 and 2. [file irsp-38-1068-s1.pdf]

Supplementary Materials

Study 1. Correlational Study

Additional Scale Information

Moral Foundations. We assessed moral foundations using the 30-item version of the Moral Foundations Questionnaire (Graham et al., 2011, adapted to Spanish by Gudiño Paredes & Fernández Cárdenas, 2015), which consists of two 15-item parts. The first part measures explicit thoughts about what is morally relevant (0 = *not at all relevant* to 6 = *extremely relevant*). The second part assesses the actual use of a moral foundation in judgement, with items rated on a scale from 0 = *strongly disagree* to 6 = *strongly agree*. The Cronbach's alphas of our study were .67, .72, .65, .72, and .69 for care, fairness, loyalty, authority, and purity, respectively. The composite of individualizing foundations was calculated by averaging the Harm and Fairness subscales (*r*[249] = .66), while the composite for binding foundations was obtained by averaging the Ingroup, Authority, and Purity subscales (with correlations between these subscales ranging from .59 to .68). Therefore, each subscale score was calculated by averaging its corresponding items, and the composite scores for individualizing and binding foundations were computed by averaging their respective subscales. A factorial analysis with Oblimin rotation yielded seven factors, as follows:

	Factor loadings						
	1	2	3	4	5	6	7
If someone suffers/or does not suffer emotionally.	-.289	**.684**		-.316		.330	-.156
If someone cares/or doesn't care about the weak and vulnerable.	-.231	**.734**	-.161	-.160	.131	.397	
If someone is/or is not cruel.	-.314	**.729**	-.248	-.260		.215	-.196
Compassion for those who suffer is the most important virtue.		**.572**	-.101	-.247		.272	.269
One of the worst things a person can do is harm a defenseless animal.		.344		**-.606**	.133		-.116
It will never be right to kill a human being.		.159			-.211	**.720**	
If someone is/or is not treated differently from others.		**.666**	-.213	-.199		.338	
If someone acts/or does not act unfairly.	-.155	**.723**	-.219	-.166			
If someone is/is not denied their rights.	-.350	**.739**	-.181	-.173	.202	.209	
When the government makes laws. the number one principle should be to make sure that all people are treated fairly.	-.247	**.755**		-.293			
Justice is the most important requirement for a society.		**.660**	.136		-.124	-.185	.141
I think it is morally wrong for a rich kid to inherit a lot of money while a poor kid inherits nothing.	-.266	.293	-.111	-.134	.440	**.553**	
If someone's action shows/or doesn't show love for her country.	**.623**	-.214	-.221		-.586		.246
If someone does/or doesn't do something that betrays their group.	.186		**-.749**		-.226	-.119	.300
If someone shows/or doesn't show a lack of loyalty.	.303	.135	**-.784**		-.195		.139
I am proud of the history of my country.	**.557**			.244	-.492	-.350	.417
People should be loyal to their family members, even if they have done something wrong.	**.546**	-.142	-.179	.313	-.195		.459
It is more important to be a team player than to play alone.			-.299	-.233		.173	**.696**
If someone shows/or does not show disrespect towards authority.	.229		-.280		**-.850**	.159	
If someone conforms/or does not conform to the traditions of society.	**.561**	-.356	-.436		-.509		.334
If an action causes/or does not cause chaos or disorder.	.282	.126	**-.594**	-.265	-.424	.129	.120

Respect for authority is something all children should learn.	.346		-.172	-.154	**-.845**		.206
Men and women have different roles in society.	**.605**	-.252			-.202	-.360	.127
If I were a soldier and disagreed with an order given by my commander and boss, I would obey it anyway, since it is my obligation to do so.	.345	-.176			-.405	-.130	**.603**
If someone violates/or does not violate the standards of purity and decency	**.596**		-.311	-.185	-.544		
If someone does/or doesn't do something unpleasant.	-.129	.238	-.447	**-.646**	-.101		.200
If someone acts / or does not act according to what God indicates or would approve.	**.727**	-.214	-.218	.112	-.315		.190
People shouldn't do things that are unpleasant. even if no one gets hurt.	.234			**-.747**	-.323		.330
I would call some acts evil. on the grounds that they are against nature.	**.710**	-.127	-.153	-.211	-.330	-.165	.227
Chastity is an important and valuable virtue.	**.777**	-.213			-.252		.147

If a five-factor solution is forced, the factor loadings are:

	Factor loadings				
	1	2	3	4	5
If someone suffers/or does not suffer emotionally.	-.371	**.707**		-.210	
If someone cares/or doesn't care about the weak and vulnerable.	-.339	**.748**	-.111		
If someone is/or is not cruel.	-.351	**.755**		-.167	
Compassion for those who suffer is the most important virtue.	-.140	**.562**	-.161	-.268	
One of the worst things a person can do is harm a defenseless animal.		.360	.108	**-.507**	
It will never be right to kill a human being.	-.195	.163			**-.525**
If someone is/or is not treated differently from others.	-.186	**.674**	-156	-.118	-.194
If someone acts/or does not act unfairly.	-.138	**.727**		-.121	
If someone is/is not denied their rights.	-.371	**.758**			
When the government makes laws. the number one principle should be to make sure that all people are treated fairly.	-.175	**.754**	.117	-.264	
Justice is the most important requirement for a society.	.211	**.618**	.130	-.122	
I think it is morally wrong for a rich kid to inherit a lot of money while a poor kid inherits nothing.	**-.514**	.325	-.180		.121
If someone's action shows/or doesn't show love for her country.	**.583**	-.254	-.323		-.547
If someone does/or doesn't do something that betrays their group.	.217		**-.718**	-.124	-.176
If someone shows/or doesn't show a lack of loyalty.	.196	.137	**-.747**		-.273
I am proud of the history of my country.	**.733**	-.161	-.146		-.242
People should be loyal to their family members, even if they have done something wrong.	**.465**	-.192	-.422	.210	-.215
It is more important to be a team player than to play alone.			**-501**	-.388	
If someone shows/or does not show disrespect towards authority.	.279		-.202	-.121	**-.826**
If someone conforms/or does not conform to the traditions of society.	.522	-.383	**-.525**	-.168	-.453
If an action causes/or does not cause chaos or disorder.	.202	.127	**-.538**	-.279	-.486
Respect for authority is something all children should learn.	.470		-.139	-.272	**-.711**
Men and women have different roles in society.	**.629**	-.291	-.172		
If I were a soldier and disagreed with an order given by my commander and boss, I would obey it anyway, since it is my obligation to do so.	**.464**	-.230	-.264	-.211	-.238
If someone violates/or does not violate the standards of purity and decency	.466		-.318	-.148	**-.600**
If someone does/or doesn't do something unpleasant.	-.121	.258	-.369	**-.682**	
If someone acts / or does not act according to what God indicates or would approve.	**.562**	-.250	-.385		-.375
People shouldn't do things that are unpleasant. even if no one gets hurt.	.199		-.175	**-.788**	-.313
I would call some acts evil. on the grounds that they are against nature.	**.655**	-.169	-.289	-.241	-.254
Chastity is an important and valuable virtue.	**.637**	-.255	-.268		-.264

Moralization of Obesity. We measured the extent to which participants morally disapprove obesity using five items used by Ringel and Ditto (2019): 1) Gaining an excessive amount of weight is disrespectful to one's body; 2) Thinness is a moral virtue; 3) Obesity is a moral failing; 4) Obesity is a sign of personal weakness; 5) If a person is capable of being thin, they should be thin. Cronbach’s alpha for this scale was .81. Importantly, the original scale was designed to measure general moral disapproval of obesity and the type of absolutist thinking characteristic of moral

attitudes. However, the three items that specifically assess absolutist thinking were not used in the present study, as they were beyond its objectives. A factorial analysis with Oblimin rotation was conducted. All items loaded on a single factor:

Item	Factor loading
Gaining an excessive amount of weight is disrespectful to one's body.	.723
Thinness is a moral virtue.	.861
Obesity is a moral failing.	.882
Obesity is a sign of personal weakness	.781
If a person is capable of being thin, they should be thin.	.615

Moral Stereotypes. To assess the moral traits attributions toward individuals with obesity, we asked participants to indicate to what extent they thought that the following traits described individuals with obesity: 1) honest; 2) fair; 3) sincere; 4) trustworthy; 5) morally committed; 6) generous; 7) with deep moral values; 8) ethically responsible. Participants rated each trait on a scale from 0 = *not at all* to 6 = *extremely*. Cronbach's alpha for this scale was .94. The first three items were drown from Leach et al. (2007), who conceptualize morality as a central component of group impression formation, nested within the broader warmth dimension (see also Abele et al., 2021) The remaining items have been used in previous research examining moral perception of ideological outgroups ("Reference to recent work by the authors, blinded for peer review"). All items reflect morally positive traits; therefore, lower scores indicate a tendency to deny moral worth, which has been theorized as one way stigmatized groups are devalued in moral terms (Brambilla et al., 2021). A factorial analysis with Oblimin rotation was conducted. All items loaded on a single factor:

Item	Factor loading
Honest.	.945
Fair.	.945
Sincere.	.980
Trustworthy.	.979
Morally committed.	.934
Generous.	.979
With deep moral values.	.961
Ethically responsible.	.969

Anti-fat Attitudes. To assess the attitudes toward people with obesity we used the 13-item Anti-fat Attitudes (AFA; Crandall, 1994, adapted to Spanish by Magallares and Morales, 2014) scale. Seven items measured dislike of people with obesity (e.g., "I don't have many friends that are fat," $\alpha = .83$), three items measured fear of fat (e.g., "I worry about becoming fat," $\alpha = .80$), and three items measured beliefs about willpower (e.g., "Some people are fat because they have no

willpower," α = .83). We added three extra items related to the disgust subscale (e.g., "people with obesity disgust me," α = .72) and taken from Quinn and Crocker's (1999) modification of the AFA scale. A factorial analysis with Oblimin rotation yielded four factors, as follows:

Item	Factor loadings			
	1	2	3	4
I really don't like fat people much.	**.633**	.383	.521	.600
I don't have many friends that are fat.	.323	.274	.435	**.476**
I tend to think that people who are overweight are a little untrustworthy.	.388	.218	.313	**.817**
Although some fat people are surely smart, in general, I think they tend not to be quite as bright as normal weight people.	.381		.313	**.717**
I have a hard time taking fat people too seriously.	.610	.298	.324	**.830**
Fat people make me somewhat uncomfortable.	**.814**	.330	.396	.665
If I were an employer looking to hire, I might avoid hiring a fat person.	.601	,307	.463	**.705**
I feel disgusted with myself when I gain weight.	.236	**.867**	.251	.163
One of the worst things that could happen to me would be if I gained 25 pounds.	.244	**.861**	.176	.280
I worry about becoming fat.	.262	**.799**	.252	
People who weigh too much could lose at least some part of their weight through a little exercise.	.355	.200	**.858**	.202
Some people are fat because they have no willpower.	.399	.242	**.875**	.335
Fat people tend to be fat pretty much through their own fault.	.475	.288	**.838**	.516
I feel repulsed when I see a fat person.	**.926**	.304	.424	.530
Fat people disgust me.	**.928**	.259	.416	.468
I have an immediate negative reaction when I meet a fat person.	**.859**	.265	.427	.373

Discrimination. We used two items adapted from Ringel and Ditto (2019) to assess discrimination against individuals with obesity: 1) People with obesity should have to pay for two seats on an airplane; 2) People with obesity should have to pay more for health insurance than thin people. Both items were significantly correlated ($r[251] = .63$, $p < .001$), so for the analysis we used the average score of both items. A factorial analysis with Oblimin rotation was conducted. All items loaded on a single factor:

Item	Factor loading
People with obesity should have to pay for two seats on an airplane.	.903
People with obesity should have to pay more for health insurance than thin people.	.903

Willingness to Participate in Collective Action for People with Obesity Rights. We assessed collective action asking participants to what extent they were willing to engage in four actions on a scale adapted from Duncan (1999) ranging from 0 = *not willing at all* to 6 = *totally willing*: 1) Sign a petition in favor of the rights of people with obesity; 2) Donate money to defend the rights of people with obesity; 3) Actively participate in an organization that defends the rights of people with obesity; 4) Attending demonstrations to demand the rights of people with obesity.

Cronbach’s alpha for this scale was .90. A factorial analysis with Oblimin rotation was conducted. All items loaded on a single factor:

Item	Factor loading
Sign a petition in favor of the rights of people with obesity.	.792
Donate money to defend the rights of people with obesity.	.861
Actively participate in an organization that defends the rights of people with obesity.	.926
Attending demonstrations to demand the rights of people with obesity.	.932

Regression Analysis

We ran eight hierarchical linear regressions analyses to examine whether individualizing and binding foundations predicted each of the following variables: the moralization of obesity and moral stereotypes (Table S1), dislike and fear of fat (Table S2), willpower and disgust (Table S3), as well as discrimination and support for collective actions (Table S4). In each regression, we controlled for the influence of BMI, self-perceived weight, and political ideology. BMI, self-perceived weight, and political ideology were included as predictors in Step 1, followed by individualizing and binding foundations in Step 2.

Table S1

Hierarchical Regressions for Moralization of Obesity and Moral Stereotypes.

	Moralization of obesity					Moral stereotypes				
	B	*SE*	β	*T*	*P*	*B*	*SE*	β	*t*	*p*
(Constant)	.07	.44		.16	.871	1.57	.55		2.85	.005
BMI	.01	.03	.03	.40	.689	.02	.03	.05	.59	.557
Sef-Perceived weight	-.02	.15	-.01	-.11	.914	.21	.18	.11	1.19	.237
Political ideology	.34	.05	.43	7.29	< .001	.04	.06	.04	.66	.513
	$F(3, 247) = 18.40$, $p < .001$					$F(3, 247) = 2.03$, $p = .110$ R $_{adjusted}$ = .012				
(Constant)	2.17	.66		3.28	.001	.29	.86		.34	.734
BMI	-.01	.02	-.02	-.26	.797	.02	.03	.05	.49	.625
Self-Perceived weight	.01	.14	.01	.07	.945	.22	.18	.11	1.24	.216
Political ideology	.18	.06	.22	3.17	.002	.06	.07	.06	.77	.447
Individualizing foundations	-.43	.09	-.27	-4.60	< .001	.22	.12	.12	1.79	.075

Binding foundations	.28	.09	.21	3.20	.002	.09	.11	.06	.80	,422
	$F(5, 245) = 18.10, p < .001$					$F(5, 245) = 2.06, p = .072$				
	$\Delta F(2, 245) = 14.60, \Delta R^2 = .087, p < .001$					$\Delta F(2, 245) = 2.05, \Delta R^2 = .016, p = .131$				

Table S2

Hierarchical Regressions for Dislike and Fear of Fat.

	Dislike					Fear of fat				
	B	*SE*	β	*t*	*P*	*B*	*SE*	β	*T*	*p*
(Constant)	.76	.39		1.96	.052	.79	.61		1.30	.194
BMI	.00	.02	.01	.14	.891	.00	.03	.01	.06	.955
Sef-Perceived weight	-.25	.13	-.17	-1.95	.053	.40	.20	.18	2.02	.044
Political ideology	.15	.04	.22	3.58	< .001	.13	.06	.13	1.98	.048
	$F(3, 247) = 7.48, p < .001$					$F(3, 247) = 3.99, p = .008$				
						$R_{adjusted} = .035$				
(Constant)	2.79	.58		4.82	< .001	2.13	.95		2.24	.026
BMI	-.01	.02	-.03	-.39	.694	.00	.03	.01	.12	.908
Self-Perceived weight	-.23	.12	-.16	-1.91	.057	.39	.20	.18	1.98	.048
Political ideology	.02	.05	.02	.30	.764	.10	.08	.10	1.30	.194
Individualizing foundations	-.40	.08	-.31	-4.91	< .001	-.23	.13	-.12	-1.73	.085
Binding foundations	.18	.08	.16	2.35	.019	-.08	.13	-.05	-.61	,543
	$F(5, 245) = 10.55, p < .001$					$F(5, 245) = 3.12, p = .009$				
	$\Delta F(2, 245) = 13.97, \Delta R^2 = .094, p < .001$					$\Delta F(2, 245) = 1.78, \Delta R^2 = .014, p = .171$				

Table S3

Hierarchical Regressions for Willpower and Disgust.

	Willpower					Disgust				
	B	*SE*	β	*t*	*p*	*B*	*SE*	β	*t*	*p*
(Constant)	.95	.63		1.50	.135	.13	.40		.33	.745
BMI	.02	.04	.06	.68	.498	.01	.02	.05	.60	.552
Sef-Perceived weight	-.23	.21	-.10	-1.09	.275	-.18	.13	-.12	-1.36	.176
Political ideology	.34	.07	.31	5.08	< .001	.13	.04	.20	3.16	.002
	$F(3, 247) = 9.84, p < .001$					$F(3, 247) = 4.59, p = .004$				
	$R^2_{adjusted} = .096$					$R^2_{adjusted} = .041$				
(Constant)	3.60	.96		3.76	< .001	2.29	.60		3.82	< .001
BMI	.01	.03	.01	.15	.885	.01	.02	.02	.25	.801

	B	SE	β	t	p	B	SE	β	t	p
Self-Perceived weight	-.20	.20	-.09	-.99	.325	-.17	.12	-.12	-1.34	.180
Political ideology	.14	.08	.13	1.72	.086	.02	.05	.03	.40	.687
Individualizing foundations	-.53	.13	-.26	-3.99	< .001	-.41	.08	-.32	-4.87	< .001
Binding foundations	.32	.13	.18	2.57	.011	.11	.08	.09	1.34	,180

	$F(5, 245) = 10.55$, $p < .001$	$F(5, 245) = 7.94$, $p < .001$
	$\Delta F(2, 245) = 10.49$, $\Delta R^2 = .070$, $p < .001$	$\Delta F(2, 245) = 12.33$, $\Delta R^2 = .087$, $p < .001$

Table S4

Hierarchical Regressions for Discrimination and Collective Action.

	Discrimination					Collective action				
	B	*SE*	β	*t*	*p*	*B*	*SE*	β	*t*	*p*
(Constant)	-.28	.55		-.52	.607	3.23	.73		4.40	< .001
BMI	.03	.03	.09	.98	.327	.03	.04	.06	.70	.482
Sef-Perceived weight	-.23	.18	-.11	-1.27	.204	-.09	.24	-.03	-.37	.710
Political ideology	.32	.06	.33	5.51	< .001	-.31	.08	-.25	-4.03	< .001
	$F(3, 247) = 11.75$, $p < .001$					$F(3, 247) = 5.53$, $p = .001$<br>$R_{adjusted} = .051$				
(Constant)	2.53	.82		3.07	.002	-1.04	1.10		-.94	.350
BMI	.02	.03	.05	.62	.534	.03	.04	.07	.84	.402
Self-Perceived weight	-.21	.17	-.11	-1.24	.215	-.09	.23	-.03	-.38	.708
Political ideology	.16	.07	.17	2.37	.019	-.16	.09	-.13	-1.76	.080
Individualizing foundations	-.54	.12	-.29	-4.65	< .001	.77	.15	.32	4.98	< .001
Binding foundations	.16	.11	.10	1.51	.132	.01	.15	.01	.09	.930
	$F(5, 245) = 12.22$, $p < .001$<br>$\Delta F(2, 245) = 11.44$, $\Delta R^2 = .075$, $p < .001$					$F(5, 245) = 8.64$, $p < .001$<br>$\Delta F(2, 245) = 12.54$, $\Delta R^2 = .087$, $p < .001$				

Study 2. Experimental Study

Additional Scale Information

Moralization of Obesity. It was measured as in Study 1. A factorial analysis with Oblimin rotation was conducted. All items loaded on a single factor:

Item	Factor loading
Gaining an excessive amount of weight is disrespectful to one's body.	.768
Thinness is a moral virtue.	.770
Obesity is a moral failing.	.833
Obesity is a sign of personal weakness	.824
If a person is capable of being thin, they should be thin.	.514

Anti-Fat Attitudes. It was measured as in Study 1. A factorial analysis with Oblimin rotation yielded four factors, as follows:

	Factor loadings			
Item	1	2	3	4
I really don't like fat people much.	**.688**	.338	.389	.326
I don't have many friends that are fat.	.452		.170	**.654**
I tend to think that people who are overweight are a little untrustworthy.	**.630**	.282	.391	
Although some fat people are surely smart, in general, I think they tend not to be quite as bright as normal weight people.	**.713**	.212	.225	
I have a hard time taking fat people too seriously.	**.773**	.247	.255	.232
Fat people make me somewhat uncomfortable.	**.767**	.267	.216	.425
If I were an employer looking to hire, I might avoid hiring a fat person.	**.696**	.308	.415	.425
I feel disgusted with myself when I gain weight.	.336	**.871**	.214	
One of the worst things that could happen to me would be if I gained 25 pounds.	.339	**.826**	.182	
I worry about becoming fat.	.190	**.852**	.190	.125
People who weigh too much could lose at least some part of their weight through a little exercise.	.168		**.801**	.223
Some people are fat because they have no willpower.	.328	.234	**.868**	.105
Fat people tend to be fat pretty much through their own fault.	.422	.343	**.789**	-.121
I feel repulsed when I see a fat person.	**.868**	.330	.260	
Fat people disgust me.	**.821**	.277	.210	
I have an immediate negative reaction when I meet a fat person.	**.851**	.335	.298	.209

Discrimination. It was measured as in Study 1. A factorial analysis with Oblimin rotation was conducted. All items loaded on a single factor:

Item	Factor loading
People with obesity should have to pay for two seats on an airplane.	.872
People with obesity should have to pay more for health insurance than thin people.	.872

Manipulation Checks. To check the effectiveness of our manipulation, we used the following five items adapted from Argüello-Gutiérrez et al. (2024): "To what extent do you find the research we told you about at the beginning on the factors that influence the perception of well-being and happiness was.… 1) convincing; 2) plausible; 3) realistic; 4) credible; 5) possible." All items loaded on a single factor:

Item	Factor loading
Convincing	.905
Plausible	.900
Realistic	.933
Credible	.940
Possible	.900

Regression Analysis

We used Hayes' (Model 1, 2017) PROCESS macro for IBM SPSS (Version 27, 2020) to test whether political ideology moderated the effect of the manipulations on moralization of obesity, dislike, fear of fat, willpower, disgust and discrimination. We therefore defined six separate moderation models to examine the moderating role of moralization of obesity on the relationship between manipulation and the dependent variable. In all the analysis we included the BMI and self-perceived weight as covariate variables. The independent variable was dummy coded: 0 = control condition; 1 = care condition; 2 = purity condition.

Regression on Moralization of Obesity. The results for moralization of obesity yielded a significant Condition × Ideology interaction (b = .122, SE = .04, p = .006; 95% CI [0.0352, 0.2087]), a nonsignificant main effect for condition (b = –.249, SE = .16, p = .123; 95% CI [–0.5655, 0.0677]), and a significant main effect for ideology (b = .133, SE = .06, p = .015; 95% CI [0.0147, 0.2520]). The Johnson-Neyman analysis revealed that the effect turned significant at the value 3.11 (b = .131, SE = .07, p = .050; 95% CI [0.0000, 0.2612]), and kept increasing until the value of 7.00 (b = .605, SE = .17, p < .001; 95% CI [0.2616, 0.9481]).

Regression on Dislike. The results for dislike yielded a significant Condition × Ideology interaction (b = .080, SE = .03, p = .019; 95% CI [0.0132, 0.1468]), a nonsignificant main effect for condition (b = – .127, SE = .12, p = .307; 95% CI [–0.3706, 0.1170]), and a nonsignificant main effect for ideology (b = .069, SE = .05, p = .137; 95% CI [–0.0221, 0.1606]). The Johnson-Neyman analysis revealed that the effect turned significant at the value 2.88 (b = .104, SE = .05, p = .050;

95% CI [0.0000, 0.2078]), and kept increasing until the value of 7.00 (*b* = .433, *SE* = .13, *p* = .001; 95% CI [0.1689, 0.6976]).

Regression on Fear of Fat. The results for fear of fat yielded a marginally significant Condition × Ideology interaction (*b* = .105, *SE* = .06, *p* = .074; 95% CI [–0.0104, 0.2211]), a nonsignificant main effect for condition (*b* = –.295, *SE* = .22, *p* = .170; 95% CI [–0.3706, 0.1170]), and a nonsignificant main effect for ideology (*b* = –.069, *SE* = .08, *p* = .394; 95% CI [–0.2271, 0.0896]).

Regression on Willpower. The results for willpower yielded a nonsignificant Condition × Ideology interaction (*b* = .040, *SE* = .05, *p* = .464; 95% CI [–0.0676, 0.1482]), a nonsignificant main effect for condition (*b* = –.003, *SE* = .20, *p* = .987; 95% CI [–0.3971, 0.3908]), and a significant main effect for ideology (*b* = .228, *SE* = .08, *p* = .003; 95% CI [0.0808, 0.3760]).

Regression on Disgust. The results for disgust yielded a significant Condition × Ideology interaction (*b* = .102, *SE* = .03, *p* = .003; 95% CI [0.0348, 0.1697]), a nonsignificant main effect for condition (*b* = –.202, *SE* = .13, *p* = .108; 95% CI [–0.4477, 0.0446]), and a nonsignificant main effect for ideology (*b* = .020, *SE* = .05, *p* = .671; 95% CI [–0.0723, 0.1122]). The Johnson-Neyman analysis revealed that the effect turned significant at the value 2.98 (*b* = .103, *SE* = .05, *p* = .050; 95% CI [0.0000, 0.2064]), and kept increasing until the value of 7.00 (*b* = .514, *SE* = .14, *p* < .001; 95% CI [0.2473, 0.7811]).

Regression on Discrimination. The results for discrimination yielded a marginally significant Condition × Ideology interaction (*b* = .108, *SE* = .06, *p* = .051; 95% CI [–0.0004, 0.2157]), a nonsignificant main effect for condition (*b* = –.213, *SE* = .20, *p* = .290; 95% CI [–0.6073, 0.1817]), and a nonsignificant main effect for ideology (*b* = .060, *SE* = .08, *p* = .426; 95% CI [–0.0808, 0.2077]).

References

Hayes, A. F. (2017). *Introduction to mediation, moderation, and conditional process analysis: A regression-based approach.* Guilford publications.

IBM Corp. (2020). *IBM SPSS Statistics for Windows (Version 27.0.)* [Computer software].
